# Melanoma-specific survival of patients with uveal melanoma and liver metastases diagnosed between 2005 and 2021

**DOI:** 10.1177/17588359241273020

**Published:** 2024-08-23

**Authors:** Lisa Wiens, Gerd Grözinger, Helmut Dittmann, Karolin Thiel, Ulrike Leiter, Teresa Amaral, Lena Nanz, Lukas Flatz, Andrea Forschner

**Affiliations:** Center for Dermatooncology, Department of Dermatology, Eberhard Karls University of Tübingen, Liebermeisterstr. 25, Tübingen 72076, Germany; Department of Diagnostic and Interventional Radiology, Eberhard Karls University of Tübingen, Tübingen, Germany; Department of Nuclear Medicine and Clinical Molecular Imaging, Eberhard Karls University of Tübingen, Tübingen, Germany; Department of General, Visceral and Thorax Surgery, Oberschwabenklinik, Ravensburg, Germany; Center for Dermatooncology, Department of Dermatology, Eberhard Karls University of Tübingen, Liebermeisterstr, Germany; Center for Dermatooncology, Department of Dermatology, Eberhard Karls University of Tübingen, Liebermeisterstr, Germany; Center for Dermatooncology, Department of Dermatology, Eberhard Karls University of Tübingen, Liebermeisterstr, Germany; Center for Dermatooncology, Department of Dermatology, Eberhard Karls University of Tübingen, Liebermeisterstr, Germany; Center for Dermatooncology, Department of Dermatology, Eberhard Karls University of Tübingen, Liebermeisterstr, Germany

**Keywords:** chemotherapy, immunotherapy, metastasis, melanoma, uvea melanoma

## Abstract

**Background:**

Uveal melanoma is the most common malignant tumor of the eye in adults. About half of the patients develop distant metastases, most commonly liver metastases (>90%). These are associated with poorer overall survival compared to patients with extrahepatic metastases.

**Patients and methods:**

In this retrospective study, patients diagnosed with metastatic uveal melanoma between January 2005 and December 2021 and treated at the Center for Dermato-oncology at the University of Tübingen, were included. The total cohort was divided into two groups. Group 1, in which the first diagnosis of metastasis was between 2005 and 2015 and group 2 with first metastasis between 2016 and 2021. Melanoma-specific survival (MSS) and progression-free survival (PFS) were calculated with the Kaplan-Meier method, test for differences was performed by the log-rank test.

**Results:**

A total of 167 patients were included in the study. Since more than 90% of patients had developed liver metastases as their first site of metastasis, we focused our analysis on patients with liver metastases. Median MSS was 28 months (95% confidence interval (CI) (22.8–33.2 months)) in patients receiving first-line liver-directed therapy (*n* = 89) compared to 10 months (95% CI (8.4–11.6 months)) for patients with first-line systemic therapy (*n* = 45). The best MSS was found in patients of group 2 and liver-directed therapy as first-line treatment. Since survival with first-line liver-directed therapy was significantly better in group 2, subsequent systemic therapies must also be considered, especially immune checkpoint inhibitors.

**Conclusion:**

This analysis revealed that MSS has improved significantly in recent years. In our analysis, first-line liver-directed therapy was associated with improved survival compared to first-line systemic therapy. Further studies are urgently needed, for example, to investigate the combination of immune checkpoint inhibition or tebentafusp with liver-specific procedures from the outset.

## Introduction

Uveal melanoma is the most common malignant tumor of the eye in adults.^1^ The majority of these tumors typically arise from the choroid, with the iris or the ciliary body being less common sites of origin.^1^ Uveal melanoma is extremely rare. The incidence in Germany is 400–500 new cases per year.^2^ The risk of disease increases with age and reaches a maximum between the sixth and seventh decade of life.^1,2^ Approximately half of patients develop distant metastases, which can occur years after successful therapy of the primary tumor.^3^ Median overall survival (OS) after initial diagnosis of metastases is about 12 months.^4^ The survival rate decreases rapidly from 52% in the first year of metastatic disease to 25% in the second and 13% in the third year.^5^ Uveal melanoma most commonly spreads to the liver (>90%). Liver metastases are associated with poorer OS compared to patients with extrahepatic metastases.^6^

Several systemic therapies have been studied in metastatic uveal melanoma, including chemotherapy, targeted therapy, and immune checkpoint inhibition (ICI). However, prior to the approval of tebentafusp, no systemic therapy had been approved for patients with metastatic uveal melanoma. Most chemotherapies resulted in objective response rates (ORR) of <5%.^7^ Similarly, blockade of the CTLA-4 (cytotoxic T-lymphocyte-associated Protein 4) or PD1 (programmed cell death protein 1) checkpoints as monotherapy has shown limited efficacy in metastatic uveal melanoma.^8,9^

The only systemic therapy that prolonged median OS to >1 year was the combination of ipilimumab and nivolumab, with ORR ranging from 12% to 18%.^10–13^ There is some evidence that the presence of not only hepatic but also extrahepatic metastases might be associated with improved ORR with combined ICI.^14,15^ However, liver metastases are known to be associated with reduced response rates to ICI, not only in uveal melanoma but also in cutaneous melanoma. In the presence of liver metastases, melanoma-specific survival (MSS) has proven to be significantly worse and the ORR for liver metastases under treatment with ICI is drastically reduced to only 3% compared to metastases at other metastatic sites.^16^

Recently, tebentafusp, a T-cell engager, was shown to improve OS as first-line therapy in metastatic uveal melanoma patients with the specific HLA type HLA-A*02:01.^17^ However, even with tebentafusp, the ORR was low and dependent on tumor burden.^18^

Considering that liver metastases are very common and prognostically relevant, liver-directed therapies are gaining more and more importance.^19,20^ The most commonly used procedures for small and single hepatic metastases are radiofrequency ablation and surgical excision. Larger single metastases (>3 cm) are usually treated with transarterial chemoembolization and, in the presence of multiple metastases, selective internal radiotherapy (SIRT) or chemosaturation can be performed.^21,22^

Chemosaturation is a technique in which the metastatic liver lobe is saturated with high doses of melphalan through an arterial catheter. Venous blood is aspirated and cleared of melphalan by an extracorporeal filtration system, minimizing systemic toxicity.^23,24^

Here we evaluated MSS of patients with metastatic uveal melanoma diagnosed in the last 17 years. In addition, the type of first-line treatment approaches were evaluated to assess their potential impact on survival outcomes.

## Materials and methods

In this retrospective study, we included patients diagnosed with metastatic uveal melanoma and treatment at the Center for Dermato-oncology at the University of Tübingen. The reporting of this study conforms to the STROBE statement.^25^ The patients were identified using the data documented in the Central Malignant Melanoma Registry. All patients included gave their written consent for the documentation and use of their clinical data for research purposes.

Inclusion criteria were: Patients with uveal melanoma who were first diagnosed with distant metastases between January 2005 and December 2021.

Metastatic patterns were assessed at the time of first metastasis and the initial treatment approach at that time was evaluated. Response was assessed by careful review of the patient’s medical records by the investigators and was documented according to RECIST criteria.^26^ The overall response rate (ORR) was calculated, including complete response (CR) and partial response (PR). The disease control rate (DCR) included CR, PR, and stable disease (SD)). As only a very small percentage (<10%) of patients had no liver metastases, only patients with liver metastases and who received first-line therapy were included in the survival calculations. MSS and progression-free survival (PFS) were calculated with the Kaplan-Meier method, test for differences was performed by the log-rank test. Furthermore, Chi-square and Fisher’s exact test were conducted to test the differences between two cohorts. In all cases, two-tailed *p* values were calculated and considered significant with values *p* < 0.05. Statistical analysis was performed using the statistical program for social sciences SPSS version 28 (IBM, New York, USA). To enable the display of patients at risk together with the survival curves, STATA version 18 (StataCorp, Texas, USA) was used, and patients that survived >1 month are displayed.

## Results

A total of 167 patients were included in the study. The study cohort consisted of 95 women (56.9%) and 72 men (43.1%). The mean age of the women at the time of initial diagnosis was 59.7 years (34–83 years, standard deviation (SD) = 11.7), and that of the men was 62.1 years (18–84 years, SD = 12.1). The right eye was affected in 47.3% and the left eye in 52.7%.

The most common local treatment for primary tumors was surgical enucleation, closely followed by radiotherapy or combined surgery and radiotherapy. The first diagnosis of distant metastases was on average 4.9 years after initial diagnosis of uveal melanoma.

**Figure 1.**
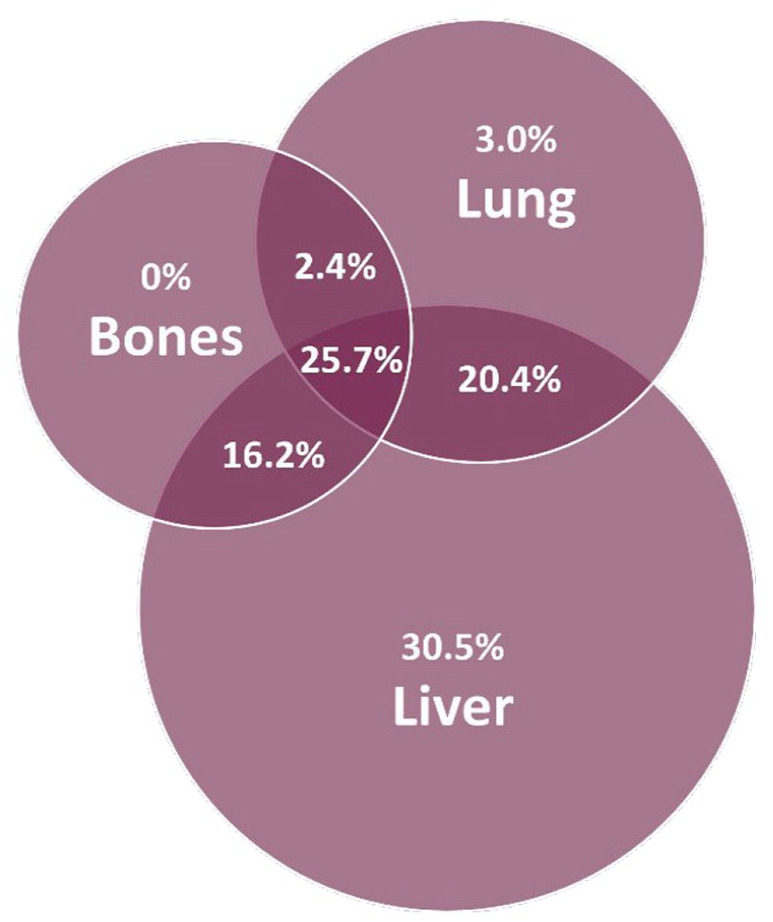
Localization of metastases.

Uveal melanoma most frequently metastasized to the liver (92.8%), lung (51.5%), and bones (44.3%). Metastases often occur in multiple organs (Figure 1).

More than half of the patients (64.7%), were followed up before the initial diagnosis of distant metastases. In 41.3%, regular radiological examinations such as magnetic resonance imaging (MRI) or computed tomography (CT) were performed before the diagnosis of distant metastases. In about 30% of patients, no follow-up radiological diagnostics such as CT or MRI were performed. For the rest of the patients, there was no documentation available on the type of follow-up. On average, the last MRI/CT scan was performed 14.4 months (2.1–164.6 months, SD = 22.7) before the initial diagnosis of distant metastases. When considering the first therapy after initial diagnosis of distant metastases, it is evident that a liver-directed therapy was used in the majority of patients (52.1%). Systemic therapy was initiated in 56 patients (33.5%) and a combination of liver-directed and systemic therapy in less than 5% of patients. All baseline characteristics are detailed in Table 1.

**Table 1.** Baseline characteristics of the total study cohort.

Parameter	Categories	Total (*n* = 167)
Age at initial diagnosis	Mean in years	60.3 (SD = 12.2)
Sex	Female	95 (56.9%)
	Male	72 (43.1%)
Side of uveal melanoma	Right	79 (47.3%)
	Left	88 (52.7%)
Treatment of primary tumor	Enucleation	80 (47.9%)
	Proton therapy	68 (40.7%)
	Combination of enucleation and proton therapy	18 (10.8%)
	No therapy	1 (0.6%)
Follow-up before distant metastases	Yes	108 (64.7%)
	No	28 (16.8%)
	Unknown	31 (18.6%)
CT/MRI before distant metastases	Yes	69 (41.3%)
	No	51 (30.5%)
	Unknown	47 (28.1%)
Sites of metastasis at time of first metastasis	Liver	155 (92.8%)
	Lung	86 (51.5%)
	Bones	74 (44.3%)
	Central nervous system	14 (8.3%)
	Skin	34 (20.3%)
	Lymph node	54 (32.3%)
	Other	69 (41.3%)
Time from diagnosis to distant metastases	Mean in years	4.9 (SD = 61.5)
ECOG performance status at time of first therapy	0	50 (29.9%)
	1	78 (46.7%)
	2	9 (5.4%)
First-line therapy	Liver-directed therapy	89 (53.3%)
	Systemic therapy	56 (33.5%)
	Combination of local and systemic therapy	8 (4.8%)
	No therapy	14 (8.4%)

CT, computed tomography; ECOG, Eastern Cooperative Oncology Group; MRI, magnetic resonance imaging.

As more than 90% of the patients had developed liver metastases as first metastatic site, and the available options for hepatic metastasis include also liver-directed therapy, we focused our evaluation on patients with liver metastases. The potential effect of any new treatment options on MSS over the years is best seen by dividing the total cohort into two groups. Group 1, in which the first diagnosis of liver metastasis was between 2005 and 2015 and group 2 with the time of the first liver metastasis diagnosis between 2016 and 2021.

While approximately half of the patients in group 1 received liver-directed therapy at the time of initial diagnosis of liver metastases, the number of patients in group 2 who received liver-directed therapy was slightly higher at 62.2%.

There were only two patients with a combination of liver-directed and systemic therapy in group 1, and six patients in group 2 received a combination treatment (3 patients received a combination of ICI and chemosaturation, 1 patient received a combination of ICI and surgery, and 2 patients combined chemotherapy and chemosaturation).

Surgery was the most common liver-directed therapy in group 1, while chemosaturation was the most common procedure in group 2.

At first staging after liver-directed therapy, the ORR in group 1 was 17.2% and the DCR was 51.7%. In group 2, the ORR was 37.3% and the DCR was 64.7%.

In group 1, 16 patients received chemotherapy as first-line systemic therapy and 6 patients received ICI (*n* = 2 ipilimumab, *n* = 4 pembrolizumab). In group 2, only 2 patients received chemotherapy as first-line systemic therapy, but 19 patients had ICI (*n* = 14 ipilimumab and nivolumab, *n* = 2 pembrolizumab, *n* = 3 nivolumab).

The first staging after initiation of systemic therapy revealed an ORR of 5.6% and a DCR of 33.3% in group 1. In group 2, the ORR was 13.3% and the DCR 46.7%.

The baseline characteristics of these groups are displayed in Table 2. With the exception of the type of initial therapy at the time of metastasis and the type of systemic therapy, there were no significant differences between the two cohorts.

**Table 2.** Baseline characteristics + first therapy of the two groups.

Parameter	Group 1 First liver metastasis 2005–2015 (*n* = 65)	Group 2 First liver metastasis 2016–2021 (*n* = 90)	*p-*value	Total cohort (*n* = 155)
Demographic characteristics				
Sex (%)			0.566^a^	
Female	*N* = 36 (55.4%)	*N* = 54 (60.0%)		*n* = 90 (58.1%)
Male	*N* = 29 (44.6%)	*N* = 36 (40.0%)		*n* = 65 (41.9%)
Age at the time of first metastasis (mean in years)	65.4 (SD = 9.1)	66.1 (SD = 12.4)	0.649^b^	65.8 (SD = 11.0)
Clinical characteristics				
CT/MRI during follow-up			0.784^a^	
Yes	*n* = 28 (43.1%)	*n* = 34 (37.8%)		*n* = 62 (40.0%)
No	*n* = 20 (30.8%)	*n* = 29 (32.2%)		*n* = 49 (31.6%)
Unknown	*n* = 17 (26.2%)	*n* = 27 (30.0%)		*n* = 44 (28.4%)
First therapy at the time of metastasis			0.249^a^	
Liver-directed therapy	*n* = 33 (50.8%)	*n* = 56 (62.2%)		*n* = 89 (57.4%)
Systemic therapy	*n* = 23 (35.4%)	*n* = 22 (24.4%)		*n* = 45 (29.0%)
Combination of local and systemic therapy	*n* = 2 (3.1%)	*n* = 6 (6.7%)		*n* = 8 (5.2%)
No therapy	*n* = 7 (10.8%)	*n* = 6 (6.7%)		*n* = 13 (8.4%)
First therapy: liver-directed treatment	*n* = 33	*n* = 56	0.336^a^	*n* = 89
RFA (Radiofrequency ablation)	*n* = 5 (15.2%)	*n* = 5 (8.9%)		*n* = 10 (11.2%)
Surgery	*n* = 10 (30.3%)	*n* = 12 (21.4%)		*n* = 22 (24.7%)
Chemosaturation	*n* = 7 (21.2%)	*n* = 21 (37.5%)		*n* = 28 (31.5%)
SIRT	*n* = 9 (27.2%)	*n* = 17 (30.4%)		*n* = 26 (29.2%)
Stereotactic radiotherapy	*n* = 2 (6.1%)	*n* = 1 (1.8%)		*n* = 3 (3.4%)
Response to liver-directed treatment	*n* = 29	*n* = 51		*n* = 80
ORR with liver-directed treatment	*n* = 5 (17.2%)	*n* = 19 (37.3%)	0.104^c^	*n* = 24 (30.0%)
DCR with liver-directed treatment	*n* = 15 (51.7%)	*n* = 33 (64.7%)	0.367^c^	*n* = 48 (60.0%)
Complete response	*n* = 4 (13.8%)	*n* = 6 (11.8%)		*n* = 10 (12.5%)
Partial response	*n* = 1 (3.4%)	*n* = 13 (25.5%)		*n* = 14 (17.5%)
Stable disease	*n* = 10 (34.5%)	*n* = 14 (27.5%)		*n* = 24 (30.0%)
Progressive disease	*n* = 14 (48.3%)	*n* = 18 (35.3%)		*n* = 32 (40.0%)
First therapy: systemic therapy	*n* = 23	*n* = 22	**<0.001**^a^ ***V*** **=** **0.589**^d^	*n* = 45
ICI	*n* = 6 (26.1%)	*n* = 19 (86.4%)		*n* = 25 (55.6%)
Chemotherapy	*n* = 16 (69.6%)	*n* = 2 (9.1%)		*n* = 18 (40.0%)
Dendritic cell vaccines	*n* = 1 (4.3%)	*n* = 1 (4.5%)		*n* = 2 (4.4%)
Response to first systemic therapy	*n* = 18	*N* = 15		*n* = 33
ORR with first systemic therapy	*n* = 1 (5.6%)	*n* = 2 (13.3%)	0.579^c^	*n* = 3 (9.1%)
DCR with first systemic therapy	*n* = 6 (33.3%)	*n* = 7 (46.7%)	0.493^c^	*n* = 13 (39.4%)
Complete response	*n* = 0	*n* = 1 (6.7%)		*n* = 1 (3.0%)
Partial response	*n* = 1 (5.6%)	*n* = 1 (6.7%)		*n* = 2 (6.1%)
Stable disease	*n* = 5 (27.8%)	*n* = 5 (33.3%)		*n* = 10 (30.3%)
Progressive disease	*n* = 12 (66.7%)	*n* = 8 (53.3%)		*n* = 20 (60.6%)
Response to first combined therapy (local and systemic therapy)	*n* = 2	*n* = 6		*n* = 8
ORR with combined therapy	*n* = 0	*n* = 2 (33.3%)	1.000^c^	*n* = 2 (25.0%)
DCR with combined therapy	*n* = 2 (100.0%)	*n* = 3 (50.0%)	0.464^c^	*n* = 5 (62.5%)
Complete response	*n* = 0	*n* = 0		*n* = 0
Partial response	*n* = 0	*n* = 2 (33.3%)		*n* = 2 (25.0%)
Stable disease	*n* = 2 (100.0%)	*n* = 1 (16.7%)		*n* = 3 (37.5%)
Progressive disease	*n* = 0	*n* = 3 (50.0%)		*n* = 3 (37.5%)

a Chi-quadrat test.

b Mann–Whitney *U* test.

c Fisher’s exact test.

d *V* = Cramér’s *V* (effect power: 0.07 small, 0.21 medium, 0.35 large).

CT, computed tomography; DCR, disease control rate; ICI, immune checkpoint inhibition; MRI, magnetic resonance imaging; ORR, objective response rates; SD, standard deviation; SIRT, selective internal radiotherapy.

**Figure 2.**
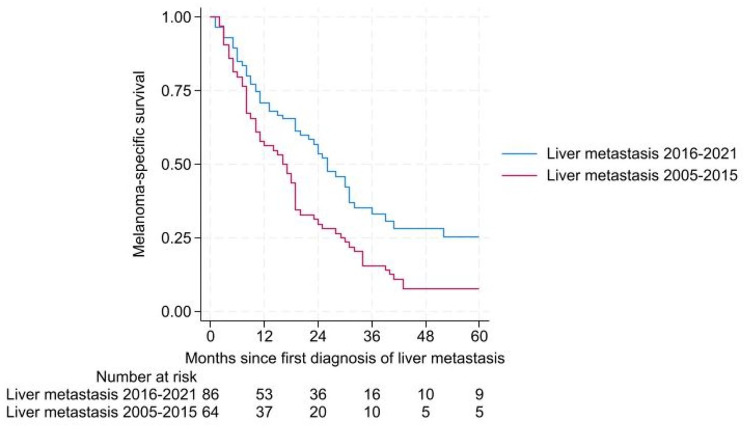
MSS of patients first diagnosed with liver metastasis 2005–2015 and 2016–2021. MSS, melanoma-specific survival.

MSS was significantly better (*p* = 0.003) in patients first diagnosed with liver metastases in 2016–2021 compared to patients first diagnosed with liver metastases in 2005–2015 (Figure 2).

The MSS of patients with hepatic metastases was significantly better in those who received a liver-directed procedure (chemosaturation, RFA, SIRT, or surgery) as initial therapy at the time of first metastases. Here, the 1-year MSS was 76.1% (95% CI (67.1%–85.1%)) and the 2-year MSS was 55.6% (95% CI (51.8%–66.2%)).

The median MSS was 28 months (95% CI (22.8–33.2 months)) in patients receiving liver-directed therapy (*n* = 89) compared to 10 months (95% CI (8.4–11.6 months)) in patients initially treated with systemic therapy (*n* = 45).

Patients treated with liver-directed therapy as initial therapy had a significantly better MSS, regardless of the time of diagnosis of liver metastases, that is, between 2005–2015 or 2016–2021.

**Figure 3.**
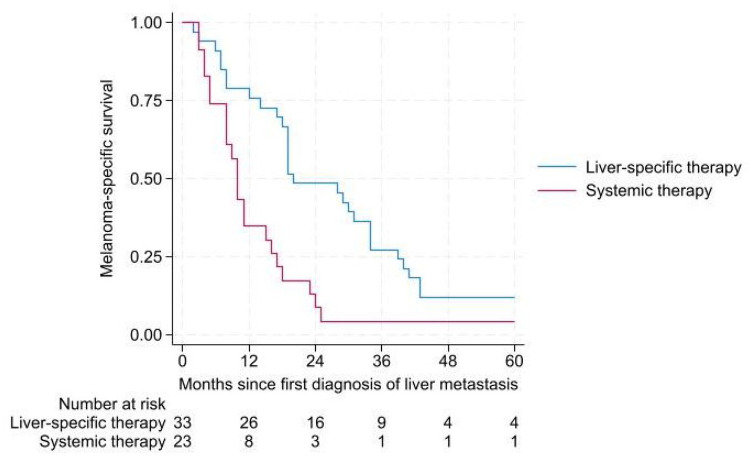
MSS in patients with diagnosed liver metastasis in 2005–2015. MSS, melanoma-specific survival.

In the 2005–2015 group, median MSS was 20 months (95% CI (12.3–27.7 months)) for patients treated with liver-directed therapy (*n* = 33) compared to 10 months (95% CI (8.5–11.6 months)) for patients receiving systemic therapy (*n* = 23) *p* < 0.001 (Figure 3).

**Figure 4.**
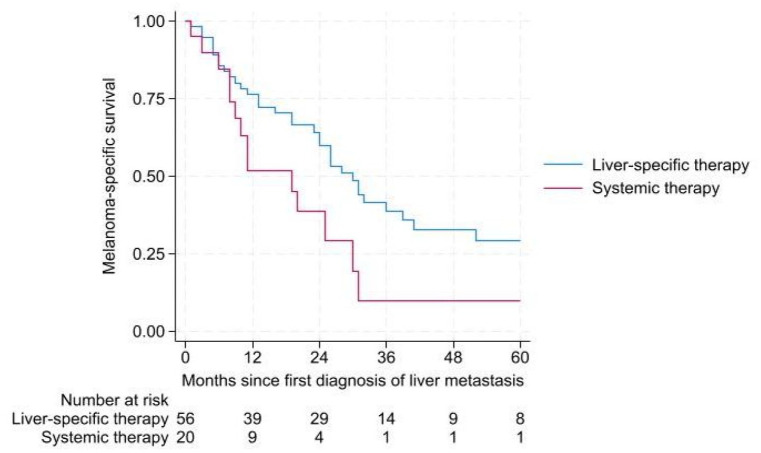
MSS in patients with diagnosed liver metastasis in 2016–2021. MSS, melanoma-specific survival.

For patients receiving liver-directed therapy in the 2016–2021 group (*n* = 56), median MSS was 30 months (95% CI (23.8–36.2 months)) compared to 11 months (95% CI (0–23 months)) for patients receiving systemic therapy (*n* = 22) *p* = 0.008 (Figure 4).

The best MSS was found in patients with liver metastases diagnosed between 2016 and 2021 and treated with liver-directed therapy as first-line treatment. Subsequent therapies are summarized in Supplemental Table 1.

## Discussion

Here we evaluated a large cohort of patients with metastatic uveal melanoma, whose clinical characteristics (sex, primary tumor localization, and age) were well balanced and consistent with the literature.^1,19^ Our analysis showed a significant improvement in MSS in patients with hepatic metastases from uveal melanoma over the last decade. The MSS was significantly better for those patients who developed liver metastases between 2016 and 2021 as compared to those from the period between 2005 and 2015.

In both groups, there was a clear survival benefit for patients who received first-line treatment with liver-directed therapy. Surgery seemed to be the liver-directed therapy associated with better survival outcomes and was superior to chemosaturation. Patients treated with SIRT had the worst outcome.

These results are comparable with those in the literature, which also demonstrate the advantages of surgery. If successful, isolated hepatectomy can prolong survival by about 18 months and is probably the most effective liver-directed therapy for metastases.^27^ Unfortunately, there are only few candidates for local hepatectomy, as an isolated metastatic lesion is rather rare. However, these results may also be biased as patients proposed for surgery are usually patients with a lower tumor burden.

Improved survival in patients with metastatic uveal melanoma treated with systemic therapy has also been shown in other retrospective studies.^4,10^ Petzold et al.^28^ concluded that the improved prognosis of metastatic uveal melanoma might probably be associated with the availability of immune checkpoint inhibitor therapies. Since 2016 it is possible to treat patients with metastatic uveal melanoma with combined ICI as at that time, combined ICI has been approved for metastatic melanoma. There is evidence in the literature that combined ICI is more effective than anti-PD-1 monotherapy, also with regard to liver metastases.^15^ Recently, a comprehensive comparison of the available systemic therapies for metastatic uveal melanoma was carried out using a complex mathematical model. Tebentafusp was associated with the longest median OS of 22 months followed by combined ICI with median OS of 16 months and anti-PD1 monotherapy with 11 months.^28^ Ipilimumab had the shortest median OS with only 8 months. Before 2015 only ipilimumab had been available to treat these patients. Therefore, the difference in MSS between the two groups in our study might be explained by the availability of more effective ICI from 2016 on.

Notably, the median MSS was 28 months for patients receiving liver-directed therapies compared to 10 months for patients treated with systemic therapy in the first-line setting. These results correspond well to data reported by Khoja et al.^29^ However, MSS for first-line liver-directed therapy was superior in group 2 compared to group 1. Therefore, subsequent systemic therapies might be responsible for a better outcome. In our cohort, additional systemic therapy provided disease control in about 35% of the patients of group 2. Of note, this subsequent therapy was ICI in all of the cases. Six patients received combined ICI with ipilimumab + nivolumab, 1 patient had been treated with pembrolizumab and another with nivolumab. In contrast, in group 1 systemic therapy given after local therapy was not associated with further disease control.

Another aspect that may explain the better outcomes in the second group could be the improvement of liver-directed therapy over the years.^30^ Nevertheless, retrospective studies still indicate that SIRT might be inferior compared to chemosaturation.^31,32^

Vogel et al.^33^ showed in their systemic review that chemosaturation is an effective regional treatment option for patients with hepatic metastases. Toxicities are transient and manageable in most cases.^33^ Hughes et al.^34^ demonstrated the efficacy of percutaneous hepatic perfusion with melphalan in a randomized phase III trial. They also found significant improvement in hepatic PFS, overall PFS, and hepatic ORR.^34^

In a retrospective study evaluating liver-directed therapies and ICI, the best outcome was seen in patients who received a combination of both. However, the rate of adverse events was high (71%), and only a very small cohort of 14 patients had been treated with liver-directed therapy plus ICI.^32^

Tebentafusp was not applied in the patients of our study as its approval was later. As recent studies have shown significantly better survival for patients with tebentafusp, further survival analyses should be performed in patients treated with tebentafusp compared to those receiving liver-directed therapy or a combination of both.

## Conclusion

This analysis revealed that MSS has improved significantly in recent years. As more than 90% of the patients had the liver as first metastatic organ, ultrasound or MRI of the liver should be implemented in the follow-up care of patients with uveal melanoma. In our analysis, first-line liver-directed therapy was associated with better survival than first-line systemic therapy. Further studies are urgently needed, for example, to investigate the combination of ICI or tebentafusp with liver-directed therapy from the outset.

## Supplemental Material

sj-docx-1-tam-10.1177_17588359241273020 – Supplemental material for Melanoma-specific survival of patients with uveal melanoma and liver metastases diagnosed between 2005 and 2021 Supplemental material, sj-docx-1-tam-10.1177_17588359241273020 for Melanoma-specific survival of patients with uveal melanoma and liver metastases diagnosed between 2005 and 2021 by Lisa Wiens, Gerd Grözinger, Helmut Dittmann, Karolin Thiel, Ulrike Leiter, Teresa Amaral, Lena Nanz, Lukas Flatz and Andrea Forschner in Therapeutic Advances in Medical Oncology

sj-docx-2-tam-10.1177_17588359241273020 – Supplemental material for Melanoma-specific survival of patients with uveal melanoma and liver metastases diagnosed between 2005 and 2021 Supplemental material, sj-docx-2-tam-10.1177_17588359241273020 for Melanoma-specific survival of patients with uveal melanoma and liver metastases diagnosed between 2005 and 2021 by Lisa Wiens, Gerd Grözinger, Helmut Dittmann, Karolin Thiel, Ulrike Leiter, Teresa Amaral, Lena Nanz, Lukas Flatz and Andrea Forschner in Therapeutic Advances in Medical Oncology

## Declarations

**Ethics approval and consent to participate:** The study is registered with the Tübingen Ethics Committee under project number 806/2022BO2. The positive ethics vote was given on December 21, 2022. The patients included gave their written consent for the documentation and use of their clinical data for research purposes.

**Consent for publication:** The patients have given their consent to publication in anonymized form.

**Availability of data and materials:** The data that support the findings of this study are available from the corresponding author, L.W., upon reasonable request.
