# Supplementary material for: Melanoma-specific survival of patients with uveal melanoma and liver metastases diagnosed between 2005 and 2021
Source: Ther Adv Med Oncol. 2024 Aug 23;16:17588359241273020. doi: 10.1177/17588359241273020 (PMC11342429; doi:10.1177/17588359241273020)
Supplement: sj-docx-1-tam-10.1177_17588359241273020 – Supplemental material for Melanoma-specific survival of patients with uveal melanoma and liver metastases diagnosed between 2005 and 2021 [file sj-docx-1-tam-10.1177_17588359241273020.docx]

1. Appendices

**Supplementary Table 1.** Course of therapy

|  | **Group 1**  **First liver metastasis diagnosis**  **2005 - 2015** | **Group 2**  **First liver metastasis diagnosis**  **2016 - 2021** | ***p-*value** | **Total cohort** |
| --- | --- | --- | --- | --- |
| Second therapy: | n = 22 | n = 60 | *p= .582* | n = 82 |
| Liver-directed therapy | n = 13 (59.1 %) | n = 27 (45.0 %) |  | n = 40 (48.8 %) |
| Systemic therapy | n = 8 (36.4 %) | n = 28 (46.7 %) |  | n = 36 (43.9 %) |
| Combined therapy (local + systemic therapy) | n = 1 (4.5 %) | n = 5 (8.3 %) |  | n = 6 (7.3 %) |
| First Staging after start second therapy (liver-directed): | n = 13 | n = 25 |  | n = 38 |
| ORR with liver-directed therapy | n = 0 | n = 8 (32.0 %) | ***p= .034^F^*** | n = 8 (21.1 %) |
| DCR with liver-directed therapy | n = 7 (53.8 %) | n = 17 (68.0 %) | *p= .486^F^* | n= 24 (63.2 %) |
| First Staging after start second therapy (systemic): | n = 7 | n = 23 |  | n = 30 |
| ORR with systemic therapy | n = 0 | n = 1 (4.3 %) | *p= 1.000^F^* | n = 1 (3.3 %) |
| DCR with systemic therapy | n = 0 | n = 8 (34.8 %) | *p= .143^F^* | n = 8 (26.7 %) |
| Third therapy: | n = 17 | n = 29 | p= .117^F^ | n = 46 |
| Liver-directed therapy | n = 3 (17.6 %) | n = 12 (41.4 %) |  | n = 15 (32.6 %) |
| Systemic therapy | n = 14 (82.4 %) | n = 17 (58.6 %) |  | n = 31 (67.4 %) |

*^C^= Chi-Quadrat-Test*

*^F^= Fisher´s Exact Test*
